# Dexamethasone-induced flares of *Trichophyton rubrum* masquerading as docetaxel cutaneous toxicity: a case report

**DOI:** 10.4076/1757-1626-2-8552

**Published:** 2009-07-23

**Authors:** Arun Azad, Melissa Kaufman, Jyotsna Jayarajan

**Affiliations:** 1 Ludwig Institute for Cancer Research, University of Melbourne, Austin HospitalAustin Health, 145-163 Studley Road, Heidelberg, VictoriaAustralia3084; 2 Department of Medicine, University of Melbourne, Austin HospitalAustin Health, 145-163 Studley Road, Heidelberg, VictoriaAustralia3084; 3 Department of Surgery, University of MelbourneAustin Health, 145-163 Studley Road, Heidelberg, VictoriaAustralia3084

## Abstract

Docetaxel chemotherapy is increasingly used in the treatment of castration-resistant prostate cancer. Cutaneous toxicity is common with docetaxel, occurring in up to 75% of cases. We present an unusual case of castration-resistant prostate cancer in which our patient developed recurrent but transient episodes of skin rash following each cycle of docetaxel. Initially, the rash was attributed to docetaxel cutaneous toxicity however a microbiological diagnosis of *Trichophyton rubrum* was subsequently made. We postulated that dexamethasone pre-medication transiently suppressed anti-fungal immunity, and indeed further flares were prevented by significantly reducing the dose of dexamethasone while continuing treatment with docetaxel.

## Introduction

Docetaxel chemotherapy is commonly used in castration-resistant prostate cancer (CRPC). Cutaneous toxicity with docetaxel is well recognised, manifesting in numerous ways including palmar/plantar erythrodysaesthesia (PPE) and nail changes (onycholysis, paronychia). We present a fascinating case in which our patient developed recurrent but transient episodes of skin rash resembling PPE following each cycle of docetaxel. Initially docetaxel cutaneous toxicity was suspected, however subsequently a microbiological diagnosis of *Trichophyton rubrum* was made. We postulated that dexamethasone pre-medication transiently suppressed anti-fungal immunity, and indeed further flares were prevented by significantly reducing the dose of dexamethasone while continuing treatment with docetaxel.

## Case presentation

A 57 year old Caucasian male with CRPC commenced docetaxel chemotherapy (75 mg/m^2^ intravenous three-weekly) and oral prednisolone (5 mg twice daily). Routine dexamethasone pre-medication (8 mg twice daily for three days starting one day pre-docetaxel) was given to prevent docetaxel hypersensitivity reactions. From cycles two to four he complained of an itchy, erythematous rash of his feet. The rash commenced one to two days after receiving docetaxel, lasted for seven to ten days, and then resolved spontaneously. He had no prior dermatological history. He did not smoke and drank minimal alcohol. He was of Macedonian origin. He lived with his wife and was retired. There was no relevant family history.

On examination, there was a sharply demarcated, erythematous, macular rash involving the soles of his feet. There was also non-tender onycholysis affecting both great toes. Nail and skin scrapings were taken with *Trichophyton rubrum* isolated on fungal culture. The patient continued on docetaxel at 75 mg/m^2^ for the next three cycles, however a single dose of dexamethasone (8 mg intravenous) was given as pre-medication one hour prior to docetaxel. The patient also commenced oral terbinafine 250 mg daily for four weeks. There were no further flares of *T. Rubrum* and no hypersensitivity reactions. Treatment was stopped after seven cycles of docetaxel because of progressive disease.

## Discussion

The time course, distribution and appearance of the rash in our patient were not helpful in conclusively differentiating docetaxel cutaneous toxicity from a fungal infection. Initially, we had suspected PPE since docetaxel is a common cause of PPE [1]. Moreover, there was a temporal relationship between the rash and treatment with docetaxel. On the other hand, this temporal relationship also existed for dexamethasone. Additionally, the absence of palmar involvement was somewhat unusual for PPE. After making a diagnosis of *T. Rubrum*, we then faced the dilemma of how best to prevent further flares while not compromising anti-cancer treatment.

We postulated that the dexamethasone pre-medication was causing transient suppression of anti-fungal immunity thereby predisposing to recurrent flares of *T. Rubrum*. Cell-mediated immunity (CMI) plays a critical role in defence against fungal infections [2]. Glucocorticoid therapy is a major risk factor for fungal infections and can impair CMI in numerous ways. This includes reduction of neutrophil and macrophage accumulation at inflammatory sites [3,4], and impaired phagocytic and microbicidal function of macrophages [5]. In addition glucocorticoids induce profound lymphopaenia, predominantly of T-cells and particularly CD4 cells. Importantly, glucocorticoids also reduce Th1 CD4 cell numbers more than Th2 CD4 cells [6]. This preferential differentiation is significant since Th1-mediated CMI is required for clearance of fungal infections [7].

Like dexamethasone, prednisolone is a glucocorticoid but it was unlikely to have contributed since it is less potent than dexamethasone. Additionally, the dose of prednisolone was low and there was no temporal relationship to the rash. We did consider the role of docetaxel, which can theoretically impair CMI since it causes neutropaenia as well as CD4 and CD8 lymphopaenia [8]. However, we felt that dexamethasone was more likely to have contributed to the flares of *T. Rubrum* and accordingly we substantially reduced the dose of dexamethasone pre-medication while maintaining the dose of docetaxel. This resulted in no further flares during three cycles of treatment, suggesting that we were correct. Our patient was also treated with oral anti-fungal therapy, which was undoubtedly important in preventing further flares.

## Conclusions

With increasing use of docetaxel for CRPC, clinicians are likely to encounter patients with skin-related problems. Given that docetaxel causes skin toxicity in as many as 75% of patients [1], docetaxel is likely to be the culprit in the vast majority of cases. Our case, while being a somewhat unusual presentation of cutaneous fungal infection, highlights the importance of considering other causes.

## Abbreviations

CMI: Cell-mediated immunity
CRPC: castration-resistant prostate cancer
PPE: palmar/plantar erythrodysaesthesia
